# Tablet Assessment of Cognition in Down Syndrome: Comparison of Researcher and Caregiver Administration

**DOI:** 10.1111/jir.70160

**Published:** 2026-08-14

**Authors:** Emily K. Schworer, Emily K. Hoffman, Anna J. Esbensen

**Affiliations:** ^1^ Waisman Center University of Wisconsin‐Madison Madison Wisconsin USA; ^2^ Division of Developmental and Behavioral Pediatrics Cincinnati Children's Hospital Medical Center Cincinnati Ohio USA; ^3^ Department of Pediatrics University of Cincinnati College of Medicine Cincinnati Ohio USA

**Keywords:** CANTAB, intellectual disability, NIH toolbox, outcome measurement

## Abstract

**Background:**

Identifying flexible and rigorous methods to administer assessments in Down syndrome research is critical for limiting participation burden. Tablet assessments have the potential to be presented remotely, but consistency between researcher and caregiver administrations is unknown.

**Method:**

Participants were 24 children with Down syndrome aged 10–17 years and their caregivers. Children completed two sessions: one administered by their caregiver and one administered by research staff. Tablet measures were selected from the NIH Toolbox and the Cambridge Neuropsychological Test Automated Battery (CANTAB).

**Results:**

There were no significant differences between child performance on researcher‐administered compared to caregiver‐administered NIH Toolbox Flanker, CANTAB RTI and CANTAB PAL measures. Floor effects impacted additional measures with high agreement (NIH Toolbox Pattern Comparison).

**Conclusions:**

Task completion did not differ across administrators, demonstrating evidence for parents' ability to facilitate assessments. Inconsistent agreement statistics warrant future work in larger samples and additional understanding of child tablet assessment performance.

Down syndrome is the most common chromosomal cause of intellectual disability, affecting approximately 1 in 707 live births per year (Mai et al. 2019). Pharmaceutical and behavioural interventions specifically targeting cognitive skills in Down syndrome are increasing, and recently there has been great progress in the psychometric evaluation of cognitive measures for children with Down syndrome administered in a laboratory or clinic setting (Pinks et al. 2023; Schworer et al. 2022).

A critical step in improving cognitive outcome assessments for children and adults with Down syndrome is to select measures that are informed by the Down syndrome neurobehavioural phenotype and that reduce participant burden (Esbensen et al. 2017). Specific strategies include selecting measures that are appropriate for the developmental level of individuals with Down syndrome, as well as employing nonverbal assessments of cognition. Computerised or tablet cognitive outcome measures have potential to fit both criteria; however, establishing measures that are feasible and demonstrate stability across multiple testing sessions is a central challenge in cognitive assessment for individuals with Down syndrome (Esbensen et al. 2017; Schworer and Esbensen 2023; Schworer et al. 2023).

Preliminary evidence supports feasibility of tablet‐based assessment of cognitive outcomes among individuals with Down syndrome. Use of the NIH Toolbox has demonstrated feasibility of a task of inhibitory control (i.e., Flanker) in groups of children and adults with Down syndrome (75%–86%) (Schworer and Esbensen 2023; Shields et al. 2020). Several measures from the Cambridge Neuropsychological Test Automated Battery (CANTAB) have also been explored to determine feasibility rates, with several subtests demonstrating acceptable feasibility (i.e., < 10% scoring at the floor on the measure) in children and young adults with Down syndrome (Edgin et al. 2017). Yet an updated version of the CANTAB Reaction Time subtest may limit that subtest to only be appropriate for those 12 years and older (Schworer and Esbensen 2023).

In addition to the need for psychometrically rigorous measures of cognitive assessments in Down syndrome, participant burden is another obstacle to implementing a successful clinical trial that warrants further investigation. Clinical trials often require multiple follow‐up visits with the family travelling to the research site. This travel results in added time and financial burden that prevent families from participating and contributes to challenges with recruitment in clinical trials (Newington and Metcalfe 2014). Previous work has validated home assessment techniques for young children with neurodevelopmental conditions to reduce travel time and cost to participate for individuals (Kelleher et al. 2020). Tablet‐based cognitive outcome measures are one tool that could be used as part of an at‐home set of assessments for monitoring intervention progress to reduce travel burden on participants, yet it is first necessary to compare child performance in researcher‐administered to caregiver‐administered testing sessions to ensure comparability of these methods.

The current study evaluated tablet assessment methods across two administrators, with the goal of establishing the basis for future tablet‐based administration at home under the supervision of a caregiver. Children were assessed at two time points to compare performance in a researcher‐administered study visit to a caregiver‐administered study visit in a clinical setting. We compared the tablet assessment performance of children and adolescents with Down syndrome administered at one session by their caregiver and at another session by a researcher. Differences were not anticipated as instructions were automated from the tablet tasks, and caregivers were given training materials to support the permitted redirection of the child during their administration of the tablet task.

## 1 Methods

### 1.1 Participants

Participants were 24 children aged 10–17 years (*M* = 12.9; SD = 2.5) with any type of documented Down syndrome diagnosis (92% trisomy 21, 8% mosaicism). All participants' primary language was English. Participants were primarily White (83.3%) and sex was split evenly (54% male, 46% female). The caregivers of the children with Down syndrome were primarily mothers (87.5%; 12.5% fathers) and aged 39–60 years (*M* = 49.8; SD = 5.6).

### 1.2 Procedures

All procedures were approved by the IRB at *the medical centre (withheld for review)*. Tablet assessments were completed with children and adolescents with Down syndrome at two time points separated by a 2‐week interval (+/−6 days). Both visits were completed at *the medical centre (withheld for review)*. At one visit, participants completed tablet cognitive assessments with trained research staff, and at the second visit, participants completed the tablet cognitive assessments supervised by their caregiver. Order of administrator‐sessions was randomised with half of the participants completing the researcher‐administered visit followed by the caregiver‐administered visit, and the other half completing the visits in the reverse order. The order of administration of the tablet cognitive assessment tasks was also randomised to further minimise order effects.

#### 1.2.1 Caregiver‐Administered Assessment

To support administration, caregivers completed a short video‐based training at the start of the session. This video‐based training reviewed allowable behaviours during the task (e.g., reminders to attend to the tablet), unallowable behaviours (e.g., giving hints to the child), and the specific instructions for each task to support caregivers' administration of tablet‐based tasks with their child. During the visit, caregivers also had access to an administration guide to assist with assessment administration. All caregiver questions were answered by research staff prior to the start of the caregiver‐administered session.

### 1.3 Measures

#### 1.3.1 Tablet Cognitive Assessments

##### 1.3.1.1 NIH Toolbox (version 2*)*

NIH Toolbox measures were selected that had a feasibility rate of 80% or higher on a sample of children with intellectual disability with a mental age above four years (Hessl et al. 2016). All NIH Toolbox assessments have set scripts for administration. Although NIH Toolbox measures are designed for ages 3–85 years, there are specific supplements for tasks designed to reduce floor effects for individuals with lower cognitive abilities. Version 2 was used, as version 3 was not available at the time of the testing sessions. Four NIH Toolbox Cognition Battery (TCB) tasks were administered: Flanker Inhibitory Control and Attention Test, Dimensional Change Card Sort Test (DCCS), Pattern Comparison Processing Speed Test and Picture Sequence Memory Test. Computed scores and age‐corrected standard scores were examined.

##### 1.3.1.2 Cambridge Neuropsychological Test Automated Battery

The CANTAB was initially recommended for use with individuals with Down syndrome (Edgin et al. 2010), yet ongoing evaluations with mixed results warrant further review of the standard administration of the CANTAB with this population (Schworer and Esbensen 2023). Scoring adjustments recommended for individuals with Down syndrome were used and included a tally of the number of stages completed and adjusted total errors based on stages completed for each task (Edgin et al. 2017). Three CANTAB assessments were administered: Paired Associate Learning (PAL), Reaction Time (RTI) and Spatial Working Memory (SWM).

#### 1.3.2 Caregiver Questionnaires

##### 1.3.2.1 Participant Characteristics

Family demographics and medical history were collected using caregiver questionnaires. Three questions were also answered by caregivers to determine their general impressions of the tablet assessments.

### 1.4 Data Analysis

The goal of the study was to compare caregiver‐administered and researcher‐administered study visits. Towards this goal, we first examined the time to complete the NIH Toolbox and CANTAB tablet measures for the caregiver‐administered and researcher‐administered cognitive tablet tasks using paired samples *t‐*tests. We also compared completion rates, floor effects and child performance for each cognitive measure between the caregiver‐administered and researcher‐administered study visits. Agreement between child performance on the caregiver‐administered and researcher‐administered tablet tasks was determined using paired samples *t‐*tests, Cohen's *d* effect size and Intraclass Correlation Coefficients (ICCs). Caregiver responses to the general impressions of the tablet assessments were reported descriptively.

## 2 Results

There were no significant differences in the time to complete the set of CANTAB measures between researchers and caregivers (M_researcher_ = 27.38 min, SD = 7.57; M_caregiver_ = 27.71 min, SD = 8.52, *p* = 0.81). On average caregivers took 5 min longer than researchers to complete the four NIH TCB measures, *t*(22) = −3.36, *p <* 0.01 (M_researcher_ = 20.78 min, SD = 4.34; M_caregiver_ = 25.35 min, SD = 5.94). Completion rates of assessments were the same between researcher‐administered and caregiver‐administered study visits (Table 1). Any non‐completion was due to a lack of understanding of the task and no responses from participants due to this lack of understanding. There were slight variations in floor effects between researcher‐administered and caregiver‐administered assessments (Table 1), with floor effects apparent in age‐adjusted standard scores.

**TABLE 1:** Completion rates of tablet assessments and number at floor for researcher‐administered and caregiver‐administered assessments.

	Completion *n* (%)	*n* at floor (researcher)	*n* at floor (caregiver)
**NIH TCB**			
Flanker computed score	23 (96%)	3/23 (13%)	0 (0%)
Flanker Age‐adjusted standard score		7/23 (30%)	8/23 (35%)
DCCS computed score	23 (96%)	4/23 (17%)	1/23 (4%)
DCCS Age‐adjusted standard score		14/23 (61%)	9/23 (39%)
Picture Sequence Memory computed score	23 (96%)	3/23 (13%)	0 (0%)
Picture Sequence Memory Age‐adjusted standard score		2/23 (9%)	3/13 (13%)
Pattern Comparison computed score	23 (96%)	0 (0%)	0 (0%)
Pattern Comparison Age‐adjusted standard score		19/23 (83%)	20/23 (87%)
**CANTAB**			
RTI Median Reaction Time (ms)	22 (92%)	0 (0%)	0 (0%)
PAL Total errors adjusted	23 (96%)	0 (0%)	0 (0%)
SWM Total errors	24 (100%)	0 (0%)	0 (0%)

Abbreviations: CANTAB = Cambridge Neuropsychological Test Automated Battery; DCCS = Dimensional Change Card Sort; PAL = Paired Associate Learning; RTI = Reaction Time; SWM = Spatial Working Memory; TCB = Toolbox Cognition Battery.

There were no significant group‐level differences in child performance between caregiver and researcher administration of the NIH TCB Flanker, NIH TCB DCCS, NIH TCB Pattern Comparison, CANTAB RTI, CANTAB PAL and CANTAB SWM (Table 2). Differences in performance were identified for the NIH Picture Sequence Memory at the group‐level (standard score 9.34 points higher on caregiver administration compared to research administration). Adequate agreement between scores of researcher and caregiver administrations was found for NIH TCB Flanker computed score, NIH TCB Pattern Comparison age‐adjusted standard scores, CANTAB RTI and CANTAB PAL (Table 2).

**TABLE 2:** Child performance on NIH Toolbox Cognition Battery (TCB) subtests and Cambridge Neuropsychological Test Automated Battery (CANTAB).

	Researcher M (SD)	Caregiver M (SD)	*t*	*p*	Cohen's *d*	ICC between researcher and caregiver [95% CI]
**NIH TCB**						
Flanker computed score	3.44 (2.61)	3.86 (2.35)	−1.16	0.26	0.24	0.76 [0.51, 0.89]
Flanker Age‐adjusted standard score	61.85 (8.55)	61.95 (8.55)	−0.05	0.96	0.01	0.40 [−0.06, 0.71]
DCCS computed score	1.48 (2.21)	1.82 (1.64)	−0.78	0.44	0.16	0.41 [0.00, 0.70]
DCCS Age‐adjusted standard score	59.21 (12.58)	58.89 (6.05)	0.12	0.91	0.03	0.34 [−0.14, 0.69]
Picture Sequence Memory computed score	345.26 (171.72)	430.90 (105.86)	−3.58	< 0.01	0.78	0.61 [0.12, 0.84]
Picture Sequence Memory Age‐adjusted standard score	76.44 (13.76)	85.78 (11.94)	−2.84	0.01	0.73	0.34 [−0.07, 0.67]
Pattern Comparison computed score	22.64 (13.78)	18.00 (12.48)	2.08	0.05	0.44	0.65 [0.32, 0.84]
Pattern Comparison Age‐adjusted standard score	58.64 (10.48)	56.73 (8.60)	1.40	0.18	0.20	0.77 [0.53, 0.90]
**CANTAB**						
RTI Median Reaction Time (ms)	573.03 (230.82)	528.37 (189.35)	1.38	0.19	0.21	0.77 [0.50, 0.90]
PAL Total errors adjusted	44.32 (21.79)	41.95 (21.56)	0.96	0.35	0.11	0.86 [0.69, 0.94]
SWM Total errors	25.96 (6.34)	25.87 (7.75)	0.04	0.97	0.01	−0.28 [−0.65, 0.17]

*Note:* ICC interpretation: poor (< 0.50), moderate (0.50–0.74), good (0.75–0.90) or excellent (> 0.90).

Abbreviations: DCCS = Dimensional Change Card Sort; PAL = Paired Associate Learning; RTI = Reaction Time; SWM = Spatial Working Memory.

Caregiver impressions of the tablet assessments were generally positive, with the majority of responses in the ‘good’ or ‘excellent’ range regarding appropriateness of assessments for children's abilities and assessments' ability to capture child strengths and task‐oriented behaviour (Table 3).

**TABLE 3:** Caregiver general impressions of tablet tasks, *n* (%).

	Poor	Fair	Good	Excellent
The appropriateness of the assessment for your child's abilities.	—	4 (16.7%)	10 (41.7%)	10 (41.7%)
The assessment's ability to capture your child's strengths.	3 (12.5%)	4 (16.7%)	8 (33.3%)	9 (37.5%)
The assessment's ability to capture your child's task‐oriented behaviour.	2 (8.3%)	2 (8.3%)	9 (37.5%)	11 (45.8%)

## 3 Discussion

The current study compared researcher‐administered and caregiver‐administered presentations of the NIH Toolbox and CANTAB tablet tasks. Task completion rates were the same for researcher‐administered and caregiver‐administered assessments. T‐test comparisons across administrator type yielded a lack of group‐level differences, which provides initial support for the ability of caregivers to function as facilitators for tablet cognitive assessments of their children. Yet, assessment of individual participant level agreement between the researcher and caregiver administrations (ICCs) were not consistent and, in some cases, a high ICC was a result of significant floor effects (e.g., NIH TCB Pattern Comparison age‐adjusted standard score). While results were mixed, it is promising that several measures had high agreement between researcher‐administered and caregiver‐administered assessments (e.g., NIH TCB Flanker computed score, CANTAB RTI and CANTAB PAL). The poor ICCs on the NIH TCB DCCS, NIH TCB Picture Sequence Memory and CANTAB SWM require additional study with a larger sample size to determine the extent to which the lack of agreement was a function of the difficulty of the task, behaviour of the adult leading the administration, or other functions of the measure.

There were also group‐level differences identified in child performance between the researcher‐administered and caregiver‐administered visits on one subtest, NIH TCB Picture Sequence Memory. This difference may indicate that the parents gave extra support in the instruction presentation or throughout this task that researchers did not. Future work should refine parent training materials to ensure fidelity of caregiver administration of this subtest as well as others identified with low ICCs between researcher and caregiver administrations. Additionally, the NIH TCB measures took slightly longer in the caregiver‐administered visit. This additional time was due to the caregivers reading the instructions aloud at slower paces. In updated versions of the NIH TCB, recordings of the directions are part of standard task administration, which would resolve this difference in administration time.

Caregiver impressions of the tablet tasks show acceptability regarding the appropriateness of assessments for child's abilities and assessments' ability to capture child strengths and task‐oriented behaviour. There were some caregivers who responded less favourably, likely due to variability in child cognition and goal‐directed behaviour. These differences in impressions of the assessments indicate a need to further develop tablet‐based and manipulative‐based cognitive assessments designed specifically for individuals with intellectual and developmental disabilities. Shortening the length of the assessment battery and simplifying instructions were two common themes in feedback from caregivers, which could be used to improve cognitive assessments for individuals with Down syndrome in the future.

Completion rates of the measures were strong (92%–100%) and in line with previous reports of the appropriateness of tablet assessments in children above the age of 12 (mean age of the current study sample; Schworer and Esbensen 2023). It is important to note, however, that this pilot study was limited in statistical power due to the small sample size and therefore results should be interpreted with caution. The small sample size also created potential instability of ICC estimates and an increased risk of Type II error. Additionally, there was potential for practice effects with the short time between sessions that could account for the modest differences in floor effects or differences in individual performance between researcher‐administered and caregiver‐administered assessments. The difference in floor effects could alternatively indicate that caregivers were providing supports or prompts beyond what is recommended and additional attention is needed to ensure caregivers are not contributing to an inflation of cognitive scores. The study also did not assess remote protocols but provided essential groundwork to establish caregiver competence in administration for future work incorporating remote procedures (e.g., ensuring fidelity checks are incorporated into remote‐assessment protocols for clearer interpretability of child performance).

While the small sample size was a limitation, this study provided preliminary evidence supporting the comparability of caregiver‐administered cognitive assessments to researcher‐administered assessments. Providing remote data collection options is essential for reducing participant burden in research, especially in clinical trials which require a high frequency of assessments. Although lab‐based assessments remain important to include in outcome monitoring, having cognitive assessments that caregivers can administer to the same standards of a researcher will provide opportunities for integrating remote monitoring into standardised research protocols. This pilot study is a first step towards increasing flexibility in research protocols through remote assessment and has the potential to increase participation from those with greater time burdens and individuals from rural communities, thus increasing the representativeness of study samples in Down syndrome research and clinical trials.

## Funding

This work was supported by the National Institute on Aging K99 AG084738, the Eunice Kennedy Shriver National Institute of Child Health and Human Development R01 HD093754 and the Jack H Rubinstein Foundation.

## Disclosure

No AI was used when writing this manuscript.

## Conflicts of Interest

The authors declare no conflicts of interest.

## Data Availability

The datasets used and analysed during the current study are available from the corresponding author on reasonable request.
